# EHMTI-0340. Distance treatment programs for individuals with complex chronic headaches living far from the hospital: the Canadian experience

**DOI:** 10.1186/1129-2377-15-S1-D16

**Published:** 2014-09-18

**Authors:** B Dick, K Reid, M Verrier, M Simmonds, S Rashiq, B Santin, L Schick, S Rogers, A Piragoff

**Affiliations:** 1 Anesthesiology and Pain Medicine, University of Alberta, Edmonton, Canada; 2 Pediatric Chronic Pain Clinic, Stollery Children's Hospital, Edmonton, Canada; 3 Clinical Telehealth Services Information Technology, Stollery Children's Hospital/University of Alberta Hospital, Edmonton, Canada

## Introduction

Individuals far from treatment centres are at increased risk of unmet needs. Given the high prevalence of headache, this is a great concern. With increased communication technology advancements including telehealth comes the opportunity to treat remote patients with limited or no access.

## Aims

1) To compare the efficacy of the Pain 101 program in headache patients who attend the program in person versus those who attend via telehealth.

2) To discuss qualitative aspects of telehealth use reported by patients and staff.

## Method

Adolescent and adult outpatients with complex headaches referred to tertiary care hospitals were enrolled in Pain 101. This pain management program targets pain education, reducing physiological arousal, goal setting and activity management, cognitive reappraisal and acceptance, and emotional regulation.

## Results

Adult and pediatric patients showed significant and lasting reductions in pain, pain-related disability, anxiety, pain-related fear, and a significant improvement in sleep and quality of life. Adults but not pediatric patients showed depression reduction. No significant differences were found between patients who attended via telehealth compared to those attending in person. Cost saving estimates to individual patients ranged from 1650–6600 Euro. Qualitatively, several patients reported that they would not have been able to attend without telehealth. Common complaints regarding telehealth were occasional technical difficulties and not feeling as involved in the group process.

## Conclusion

Strong support for telehealth technology was found for individuals with complex headaches who live far from treatment centres. Recommendations for effective use of this technology for providing headache treatment will be discussed.

No conflict of interest.

